# Leveraging omics to understand the molecular basis of acute-on-chronic liver failure

**DOI:** 10.1515/almed-2021-0023

**Published:** 2021-08-11

**Authors:** Joan Clària

**Affiliations:** Biochemistry and Molecular Genetics Service, Hospital Clínic – IDIBAPS, Barcelona, Spain; Department of Biomedical Sciences, School of Medicine and Health Sciences, Universitat de Barcelona, Barcelona, Spain

**Keywords:** advanced liver disease, bioactive lipid mediators, immunometabolism, immunosuppression, systemic inflammation

## Abstract

Acute-on-chronic liver failure (ACLF) is a complex syndrome that develops in patients with acutely decompensated cirrhosis. In this condition, dysbalanced immune function and excessive systemic inflammation are closely associated with organ failure and high short-term mortality. In this review, we describe how omic technologies have contributed to the characterization of the hyperinflammatory state in patients with acutely decompensated cirrhosis developing ACLF, with special emphasis on the role of metabolomics, lipidomics and transcriptomics in profiling the triggers (pathogen- and damage-associated molecular patterns [PAMPs and DAMPs]) and effector molecules (cytokines, chemokines, growth factors and bioactive lipid mediators) that lead to activation of the innate immune system. This review also describes how omic approaches can be invaluable tools to accelerate the identification of novel biomarkers that could guide the implementation of novel therapies/interventions aimed at protecting these patients from excessive systemic inflammation and organ failure.

## Acute-on-chronic liver failure (ACLF)

ACLF is a severe syndrome resulting in high short-term mortality evolving in patients with acutely decompensated (AD) liver cirrhosis. ACLF is defined by organ dysfunction and failures in liver, kidney, brain, coagulation and respiratory and circulatory systems, which are defined according to the criteria of the European Association for the Study of the Liver–Chronic Liver Failure (EASL‐CLIF) Consortium [1], [2], [3]. ACLF is classified into three grades of severity (ACLF-1, -2 and -3) according to the number of organ failures. As stated in the EASL‐CLIF Consortium criteria, ACLF grade 1 includes patients with single kidney failure, patients with single liver, coagulation, circulatory or lung failure associated with creatinine levels ranging from 1.5 to 1.9 mg/dL or hepatic encephalopathy grade I or grade II or both, and patients with single brain failure with creatinine levels ranging from 1.5 to 1.9 mg/dL. ACLF grade 2 includes patients with two organ failures. ACLF grade 3 includes patients with three or more organ failures. The CANONIC study, a prospective observational investigation in 1,343 patients hospitalized for acute decompensation of cirrhosis, provided the first evidence-based definition of ACLF, which includes the presence of organ failure(s) and a 28-day mortality risk of 15% or higher [1]. In Western countries, ACLF is particularly prevalent among young patients with alcohol-related liver disease, and in 60% of the cases, it develops in close association with potential precipitating events, mainly bacterial infection or active alcoholism. In Asian countries, ACLF is more commonly diagnosed in patients with hepatitis B-related cirrhosis who present lower prevalence of extrahepatic organ failures.

## Systemic inflammation is a hallmark of ACLF

Recent advances in understanding the pathophysiology of ACLF have identified an association between systemic inflammation and organ injury in patients with AD cirrhosis developing ACLF [4], [5], [6]. This systemic hyperinflammatory state is produced by the massive release of inflammatory mediators such as cytokines, chemokines, growth factors and bioactive lipid mediators with potential to induce immune-mediated tissue damage, a process known as immunopathology. For example, in the microvasculature of vital organs, proinflammatory cytokines damage the endothelium glycocalyx and trigger neutrophil and monocyte adhesion to endothelial cells and their transmigration into tissues [7]. Activated immune cells, in turn, release mediators such as proteases, oxidative molecules, cytotoxic cytokines and pro-inflammatory lipid mediators (i.e., prostaglandins [PGs] and leukotrienes [LTs]), which further intensify tissue damage.

**Figure 1:**
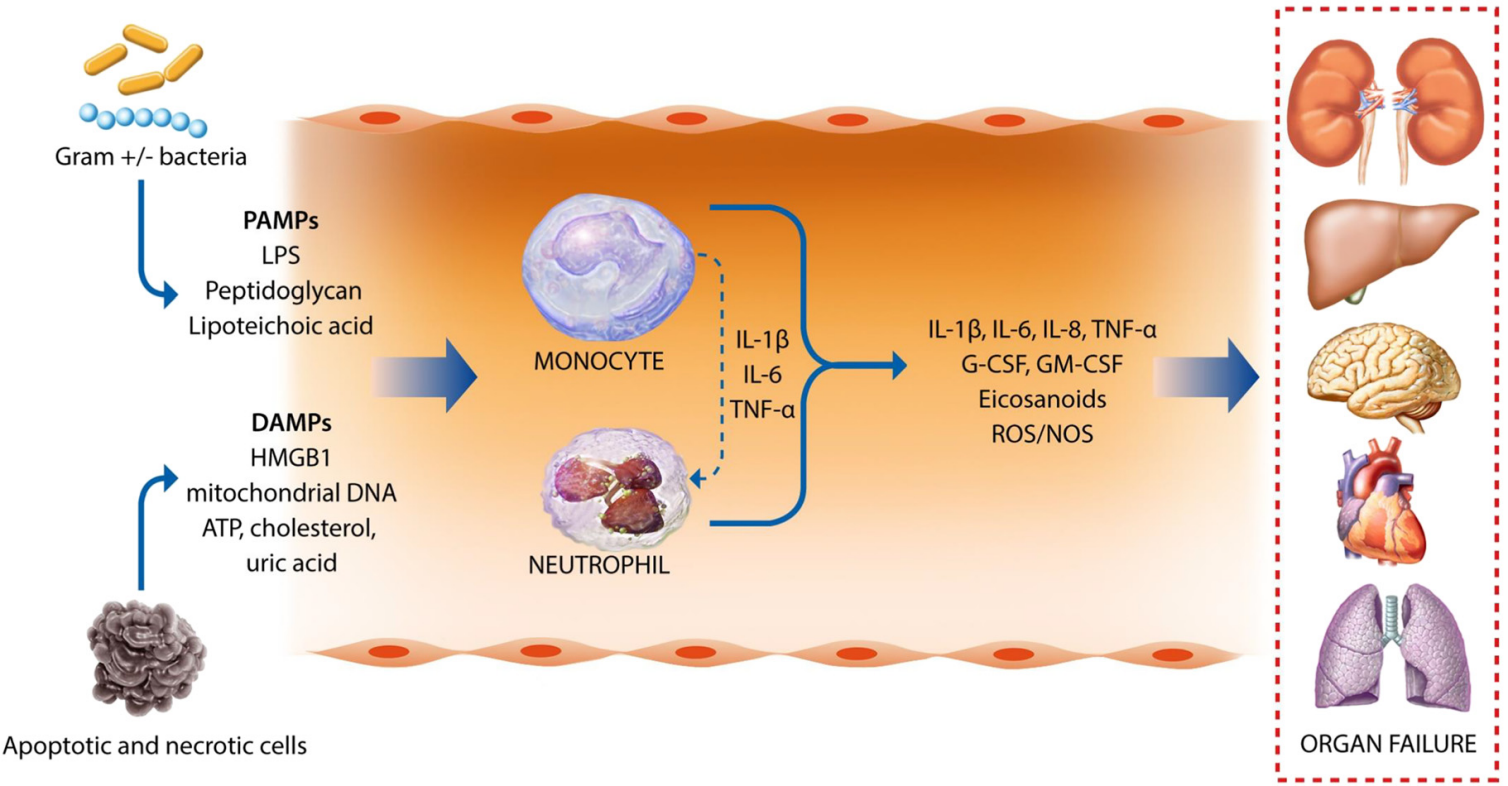
Schematic diagram summarizing the role of pathogen-associated molecular patterns (PAMPs) released by Gram+/− bacteria (lipopolysaccharide [LPS], peptidoglycan and lipoteichoic acid) and damage-associated molecular patterns (DAMPs) derived from necrotic or apoptotic dying cells (high mobility group box 1 protein (HMGB1), fragments of mitochondrial DNA, histones, ATP, cholesterol and urate crystals).

At present, little is known about the identity of the triggers (either of infectious or noninfectious origin) leading to excessive systemic inflammation in patients with AD cirrhosis evolving to ACLF. Bacterial infections are present in 33% of cases of ACLF [8] and therefore pathogen-associated molecular patterns (PAMPs) released by infecting bacteria likely contribute (Figure 1). In addition, circulating PAMPs can be the result of the translocation of bacterial products from the intestinal lumen to the systemic circulation. In fact, bacterial overgrowth, increased permeability of the intestinal mucosa and impaired function of the intestinal innate immune system are common features in AD patients developing ACLF [9]. PAMPs are uniquely conserved molecular structures that are recognized by the host via dedicated receptors called pattern-recognition receptors (PRRs), including, among others, Toll-like receptors (TLRs) present at the cell surface or in the endosomal compartment and NOD-like receptors, present in the cytosol of immune cells [10]. These receptors recognize nucleic acids and protein, lipid and carbohydrate components characteristic of bacteria and viruses. The engagement of PRRs results in the stimulation of signaling cascades that activate transcription factors such as nuclear factor (NF)-κB or activator protein-1 (AP-1) [11], which, in turn, induce the expression of a battery of genes encoding for molecules involved in inflammation (i.e., interleukin [IL]-6 and tumor necrosis factor [TNF]-α). Systemic inflammation can occur in patients with AD cirrhosis and ACLF in the absence of bacterial infections and/or bacterial translocation as the result of the release of damage-associated molecular patterns (DAMPs) from injured organs and tissues (Figure 1). DAMPs are released by dead, dying or injured cells and originate from several cellular compartments, especially from the nucleus (high mobility group box 1 [HMGB1] and histones), mitochondria (mitochondrial DNA and formyl peptides) and the cytosol (adenosine triphosphate [ATP]) [11].

The systemic hyperinflammatory response in patients with ACLF frequently occurs in parallel with the presence of a dysfunctional innate immune system at the humoral, physical and cell-mediated level [6, 12, 13]. Due to hepatocellular insufficiency, cirrhotic patients commonly display reduced humoral anti-defense capacities as a result of decreased production of acute phase proteins, hypoalbuminemia and a defective complement system [14, 15]. Additionally, the physical barrier in AD cirrhosis is impaired, and even more so in ACLF, with gut leakage and dysfunction of the vasculature and sinusoidal endothelium being the most prominent features [6]. Taking all these components of the innate immune system into account, the overall immune status in patients with ACLF ranges within the spectrum from immunosuppressive/immunoregulatory/tolerogenic to vigorously hyperinflammatory, and these extremes are not mutually exclusive.

## Soluble mediators of inflammation in ACLF

As described above, the dysfunctional innate immune system in patients with ACLF is characterized by increased circulating levels of small proteins (cytokines, chemokines and growth factors) and small bioactive lipids (lipid mediators) that signal exuberant inflammatory and immune responses.

### Multiplex analysis of plasma cytokines, chemokines and growth factors

Cytokines are low-molecular-weight glycoproteins that orchestrate the effectiveness of innate immunity by inducing local inflammation and acute systemic responses [16, 17]. The production of cytokines by leukocytes is one of the initial steps of the inflammatory cascade. Once released, cytokines bind specific receptors in their target cells [18]. Although leukocytes are the major source of cytokines, parenchymal cells are increasingly recognized as contributing to the production of inflammatory cytokines and interacting with leukocytes to optimize immune responses [19]. Cytokines are also important in initiating, amplifying and mediating adaptive immunity [20]. Cytokines can be classified into different families including TNF, IL-6 and interferon (IFN) α, β, and γ families. Cytokines can also be categorized according to their role as pro-inflammatory or anti-inflammatory mediators. TNF-α, IL-1β and IL-6 are well-characterized pro-inflammatory cytokines, whereas IL-4, IL-10 and IL-1 receptor antagonist (IL-1ra) are considered anti-inflammatory [20]. The presence of increased circulating levels of TNF-α and IL-6 in patients with cirrhosis without infections was described decades ago [21, 22], but comprehensive characterization of these inflammatory mediators in ACLF and their correlation with disease severity and organ failures were described more recently [4, 5]. In this context, a major cytokine is IL-6, which is a pleiotropic cytokine produced in response to infections and tissue injuries. IL-6 synthesis and secretion are induced upon stimulation of TLR4 by lipopolysaccharide (LPS), IL-1β or TNF-α and is one of the major stimuli for the release of hepatic acute phase proteins. IL-6 strongly correlates with the development of renal impairment and mortality in patients with cirrhosis and bacterial peritonitis [23]. An important aspect to consider is that in patients with ACLF the circulating levels of not only IL-6 but most cytokines are of similar magnitude to those reported in patients with sepsis. Therefore, the term “cytokine storm”, which defines the exacerbated production of these mediators as a consequence of an overactivated immune system accompanied by systemic inflammation, commonly observed in sepsis-like diseases [16], is also pertinent to ACLF.

### Analysis of circulating levels of small bioactive lipid mediators by targeted lipidomics

Bioactive lipid mediators are derived from structural lipid species (i.e., phospholipids containing polyunsaturated fatty acids [PUFA]), which compose the lipid bilayer of cell membranes [24]. Most lipid mediators are derived from omega-6 and omega-3 PUFAs, which are released on demand in response to an inflammatory stimulus from the cell membrane into the cytosol by phospholipase A_2_ [24–26]. In the cytosol, PUFA are readily converted by cyclooxygenase (COX), lipoxygenase (LOX) and cytochrome P450 (CYP) enzymatic pathways into an array of biologically active lipid mediators, which are released to exert their actions as autacoids [24–26]. The essential omega-6 PUFA arachidonic acid (AA) is the substrate for the intracellular biosynthesis of one of the main families of lipid mediators: the eicosanoids. The eicosanoid family consists of PGs, thromboxane A_2_ (TXA_2_), LTs, lipoxins (LXs) and epoxyeicosatrienoic acids (EETs). With the exception of LXs, eicosanoids have pro-inflammatory properties and, in fact, PGs and TXA_2_ are the prime targets for non-steroidal anti-inflammatory drugs (NSAIDs) [27, 28]. Similar to cytokines, eicosanoids are massively released by leukocytes in response to infections or tissue injury originating the so-called “eicosanoid storm” [24].

Eicosanoids, in particular prostaglandin E_2_ (PGE_2_), play a key role in the development of the five cardinal signs of inflammation: edema, erythema, pain, fever and loss of function. PGE_2_ increases vascular permeability contributing to fluid extravasation and the appearance of edema (swelling) [29]. In addition, PGE_2_ sensitizes peripheral sensory nerve endings located at the site of inflammation and acts in the spinal cord to evoke hyperalgesia and pain [29]. PGE_2_ is also crucial for the appearance of fever, being pyresis the consequence of increased levels of PGE_2_ in the central nervous system secondary to the actions of IL-1β produced by activated immune cells in the systemic circulation [30]. Finally, PGE_2_ has widespread immunosuppressor roles depending on its site of action and formation and has been shown to be a key mediator of myeloid-derived cell dysfunction inhibiting nicotinamide adenine dinucleotide phosphate (NADPH) oxidase-mediated bacterial killing and FcγR-mediated phagocytosis [31, 32]. On the other hand, LTs are intricately involved in allergic and inflammatory reactions and are highly potent mediators of inflammation. For example, LTB_4_ induces endothelial adhesion, chemotaxis and activation of leukocytes, lysosomal enzyme secretion and superoxide anion production in neutrophils [33, 34]. In addition, a stimulatory role for LTB_4_ on cytokine synthesis has been reported [34]. LTC_4_/LTD_4_/LTE_4_ are potent eosinophil chemoattractants, cause plasma leakage from post-capillary venules, enhance mucus secretion and induce synthesis and release of pro-inflammatory mediators including IL-8 and platelet activating factor [26, 34].

Similar to the omega-6-PUFA AA, the omega-3-PUFAs eicosapentaenoic (EPA) and docosahexaenoic (DHA) acids are also converted by the COX, LOX and CYP pathways into bioactive lipid mediators, but in this case carrying potent anti-inflammatory and pro-resolving actions [35–38]. These mediators are generically known as “specialized pro-resolving mediators” or SPM which have attracted much attention in recent years because they do not only act as “braking signals” of unremitting inflammation but also play critical roles in the dynamic resolution of tissue inflammation [35, 38]. In particular, COX and LOX enzymes can generate the SPMs known as resolvins, a term that is derived from resolution phase interaction products, which can be classified as resolvins of the D-series if they are generated from DHA or resolvins of the E-series if the biosynthesis is initiated from EPA [35, 36]. Alternatively, LOX enzymes can convert DHA into another category of SPMs designated as protectins (PD1) and maresins (MaR1 and MaR2) [35, 36]. Finally, the omega-3 PUFA docosapentaenoic acid (DPA) can also give rise to resolvins of the 13-series (RvTs) [39].

SPMs have dual roles as stop-signals for inflammation and activators of resolution of inflammation [35, 36]. The anti-inflammatory properties are specific for each SPM. For example, RvE1 decreases neutrophil infiltration and T cell migration, reduces TNF-α and IFNγ secretion, inhibits chemokine formation and blocks IL-1β-induced NF-κB activation [40]. RvD1 and RvD2 reduce inflammatory pain, block IL-1β expression induced by TNF-α and limit polymorphonuclear leukocyte (PMN) infiltration into inflamed brain, skin and peritoneum [41, 42].

RvD2, in particular, has been shown to be a potent endogenous regulator of excessive inflammatory responses in mice with microbial sepsis [43]. In addition, RvD2 has been shown to down-regulate IL-1β expression, and to reduce ASC speck formation (a proxy of inflammasome assembly) and secretion of mature IL-1β in peritoneal and bone marrow-derived macrophages [44]. On the other hand, PD1 and MaR1 exert protective actions in acute models of inflammation by blocking PMN migration and infiltration into the inflammatory site [35, 45]. In addition to their anti-inflammatory properties, SPM exert potent pro-resolving actions and expedite the resolution process also within the nanomolar range. In general, SPMs pave the way for monocyte differentiation into phagocytosing macrophages, facilitating the removal of dead or dying cells as well as bacterial clearance [46]. For example, RvE1 stimulates macrophage phagocytosis of apoptotic PMN and is a potent counter-regulator of L-selectin expression [40, 47]. Interestingly, macrophage phagocytosis of apoptotic cells also leads to the biosynthesis of SPMs, which act in an autocrine manner to facilitate phagocytosis [48].

Until recently little was known about the role of lipid mediators in ACLF. Blood levels of fatty acids have been reported to be increased in patients with AD cirrhosis and ACLF [49], although among the fatty acid repertoire, the total pool of PUFAs is invariably reduced in patients with ACLF [50]. This suppression is seen when measuring the total blood content, which more accurately represents the actual PUFA pool in the circulation and is not appreciated when the analysis only considers free circulating PUFA [51]. Furthermore, patients with ACLF exhibit a remarkable unbalance between omega-6 and omega-3 PUFA families (higher AA [omega-6] to EPA [omega-3] ratio), which is a surrogate marker of systemic inflammation and/or impaired resolution [50]. Consistent with the view of impaired resolution, in the late 90s, our group described a deficit in LXA_4_ formation in coincubations of PMN and platelets from patients with AD cirrhosis in comparison to those from healthy subjects [52]. In addition, a defective chemotactic response to LTB_4_ was identified in PMN from these patients, an abnormality that likely contributes to the characteristic bacterial killing dysfunction present in this condition [52]. O’Brien et al. provided evidence that PGE_2_ plays a critical role in driving the immunosuppressive status of patients with AD cirrhosis and ACLF, a feature that puts these patients at higher risk of recurrent infections [53]. By performing targeted lipidomics in 49 patients of the ATTIRE trial, the same group of investigators also explored the potentiality of human serum albumin infusions to restore immune function in patients with AD cirrhosis and ACLF, the effects of albumin on PGE_2_ binding and its potential interaction with proresolving lipid mediators [54]. The most relevant finding of this study was that patients with AD cirrhosis can be categorized into two distinct phenotypes according to their lipid mediator profile. One group exhibited a hypoactivated profile with reduced concentrations of several pro-resolving and inflammation-initiating mediators including PD1, LXA_4_, PGs, TXB_2_ and LTB_4_, whereas the other showed a hyperactivated status with increased production of these lipid mediators [54]. The latter group had an elevated white blood cell count and higher temperature and cytokine and C reactive protein (CRP) levels accompanied by increased plasma LPS concentrations. These investigators also identified a distinct trend between inflammation-initiating and resolution pathways between surviving and non-surviving patients with AD cirrhosis, indicating that patient survival is also associated with a shifted profile in the levels of SPMs [55]. Further studies are needed to determine the pathophysiological significance of these findings and if they can be useful to monitor the response to therapy in clinical practice.

**Figure 2:**
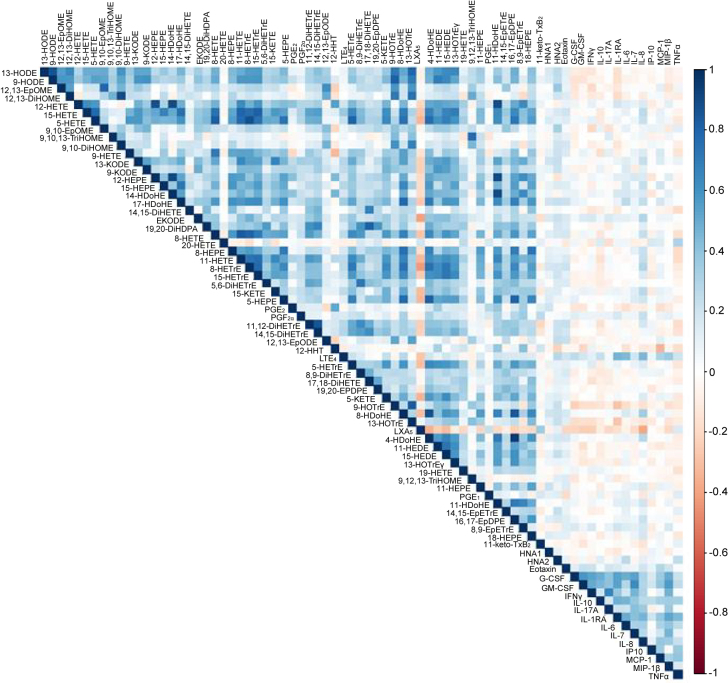
Correlation matrix plot between lipid mediators and inflammatory markers in patients with acutely decompensated cirrhosis. Data extracted from Ref. [50].

In view of the magnitude and diversity of the lipid mediator network, proper characterization of these molecules in complex diseases such as ACLF requires omics approaches in studies including a large number of patients, such as, in the CANONIC cohort. Recently, we performed a targeted lipidomics investigation using liquid chromatography coupled to tandem mass spectrometry (LC-MS/MS) to screen 100 lipid mediators in 246 patients with AD cirrhosis, 119 of whom were from the CANONIC study and had ACLF [50]. Fifty-nine lipid mediators (out of 100) were detected in plasma from cirrhotic patients and an integrated analysis revealed that ACLF was associated with higher circulating levels of LTs, PGs, epoxy-keto fatty acids and TX families, in parallel with reductions in LXs and epoxy fatty acids. Out of the 59 annotated lipid mediators, 16 were significantly associated with the disease status. Among these, 11 lipid mediators distinguished patients at any stage (with or without ACLF) from healthy subjects, whereas two lipid mediators LTE_4_ and 12-hydroxyheptadecatrienoic acid (12-HHT), both derived from AA, shaped a minimal plasma fingerprint that discriminated patients with ACLF from those without. Moreover, LTE_4_ was one of the top differentially regulated lipid mediators and gradually increased from healthy subjects to AD and ACLF, as well as in ACLF-3 as compared with ACLF-1 and -2. Interestingly, plasma levels of LTE_4_ followed the clinical course of the disease (increased with worsening and decreased with improvement). Moreover, LTE_4_ was strongly correlated with the inflammatory chemokine IL-8 (Figure 2) and the necrosis/apoptosis marker keratin 18 (K18). On the other hand, at present, the biological role of 12-HHT, which is biosynthesized by TXA_2_ synthase in an equimolar ratio to TXA_2_, remains unknown. In the past, 12-HHT was considered a mere byproduct of the biosynthesis of the potent vasoconstrictor TXA_2_, although recent studies indicate that this lipid mediator can induce chemotaxis of immune cells by binding to LTB_4_ receptor 2 [56]. Our targeted lipidomics study also detected elevated levels of the pro-inflammatory and vasoconstrictor eicosanoid PGF_2α_ in ACLF patients [50]. PGF_2α_ is widely distributed and is one of the more abundant PGs formed at sites of inflammation, where it exhibits various biological activities, including contraction of bronchial and vascular smooth muscle and regulation of renin secretion and arterial blood pressure [57]. Furthermore, PGF_2α_ was identified as a lipid mediator with the ability to distinguish patients with ACLF from healthy subjects and was the only lipid mediator associated with circulatory failure in patients with ACLF.

In contrast to LTE_4_ and PGF_2α_, LXA_5_ was found invariably reduced in patients with ACLF [50]. LXA_5_ is an EPA (omega-3 PUFA)-derived SPM that promotes the timely resolution of inflammation. Biosynthesis of LXA_5_ from endogenous sources of EPA is initiated by 15-LOX and mainly occurs in cells bearing 15-LOX activity, such as those of the immune system. Since circulating LXA_5_ levels were suppressed in patients with ACLF, in whom EPA levels were not different from those of patients with AD and healthy subjects, this observation likely reflects reduced 15-LOX rather than limited access to its biosynthetic substrate (i.e., EPA). Indeed, the analysis of gene expression in leukocytes confirmed a remarkable down-regulation of 15-LOX expression in patients with ACLF in comparison to those with AD [50]. Of interest, plasma LXA_5_ levels correlated negatively with IL-8 (Figure 2) and the necrosis/apoptosis marker K18, and together with 12,13-epoxy-9-keto-10(trans)octadecenoic acid (EKODE) (derived from the omega-6 PUFA linoleic acid) formed a signature profile associated with coagulation and liver failure in patients with ACLF [50]. Other lipid mediators derived from linoleic acid, namely 9(10)-epoxy-9Z-octadecenoic acid (EpOME) and 12(13)-EpOME, which are indicators of effective bactericidal activity, were also remarkably suppressed in ACLF [50]. Taken together, this study described unbalanced formation between pro-inflammatory (i.e., LTE_4_) and anti-inflammatory/pro-resolving (i.e., LXA_5_) lipid mediators as a hallmark of the exuberant systemic inflammation present in patients with ACLF. Moreover, this targeted lipidomics study uncovered specific lipid mediator signatures associated with severity and prognosis of patients with ACLF.

## Immunometabolism and systemic inflammation in ACLF

**Figure 3:**
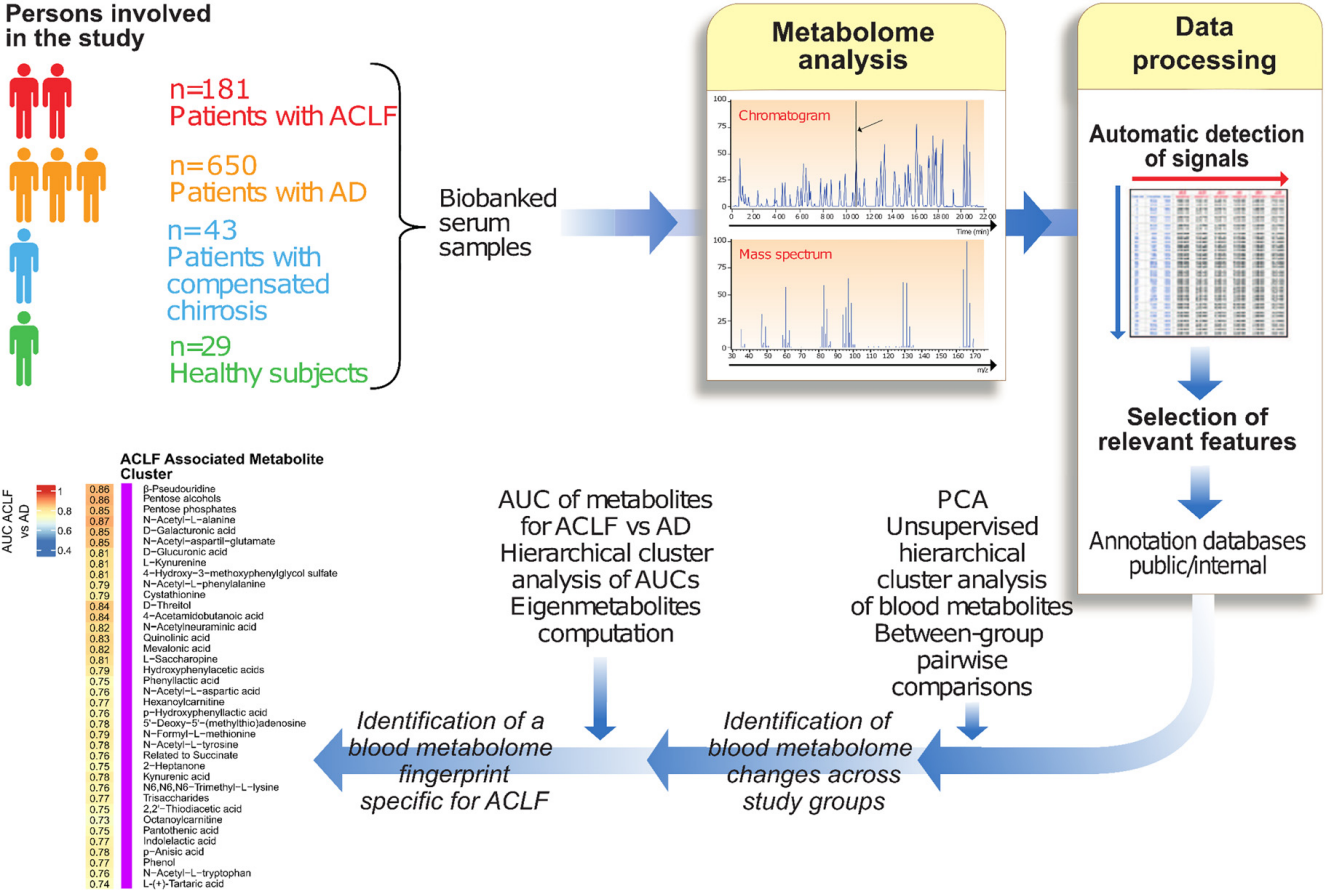
Strategy used in the metabolomics study for analyzing the blood metabolome in patients with acutely decompensated cirrhosis with and without ACLF. The data from this study are reported in Ref. [49].

Inflammatory responses are energetically expensive, requiring mobilization of stored nutrients to fuel immune activation, especially in conditions characterized by an exuberant systemic inflammation such as sepsis [58, 59]. Metabolomics, which identifies and quantifies small-molecule metabolites (the metabolome), is the omics closest to clinical phenotypes [60]. Metabolites not only reflect the metabolic activity of tissues, but because many metabolites have powerful biological activity on critical pathophysiological processes, they can also influence the clinical phenotype. The best approach to characterize the metabolomic changes of such complex syndromes as ACLF is to perform high throughput untargeted (agnostic) studies in large series of patients. Three recent studies using this approach have been published. The first is a study by Bajaj and collaborators who performed analyses in 602 patients of the NACSELD consortium [61]. The most interesting finding of this study was the identification of metabolites of microbial origin that were associated with the development of ACLF. Another study by Zacherini et al. highlighted the importance of metabolites derived from proteolysis and amino acid catabolism in ACLF [62]. Finally, the third study by Moreau and collaborators [49] included more than 831 patients prospectively enrolled in the CANONIC study (Figure 3), and the main results are described in the following paragraphs.

**Figure 4:**
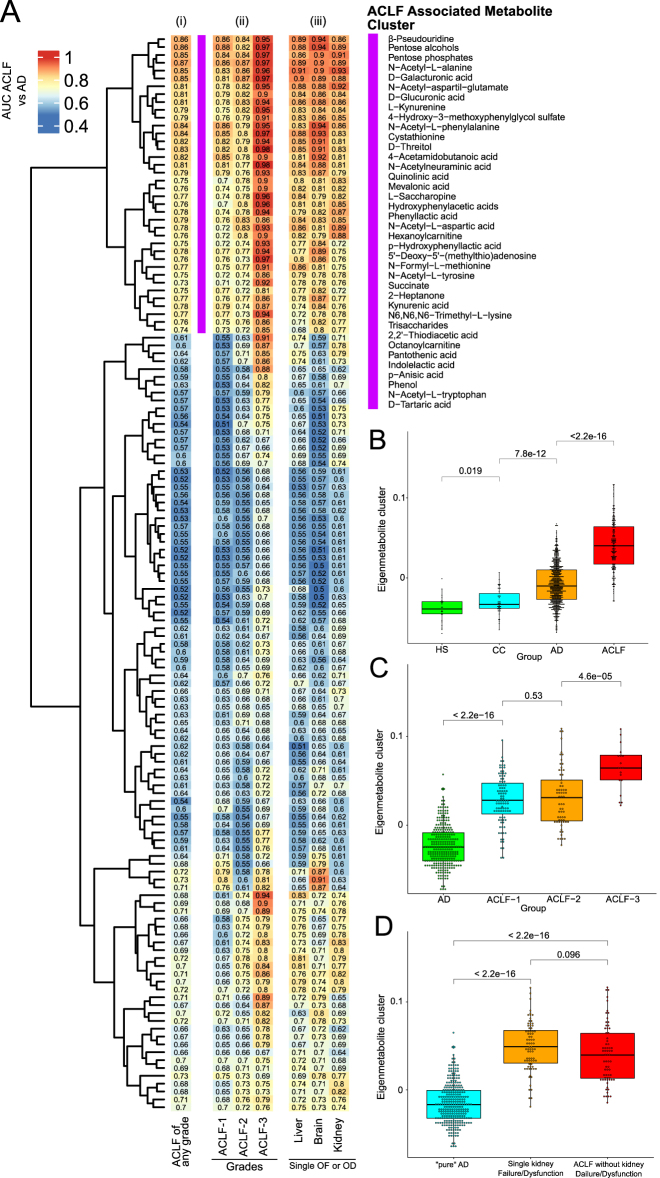
Metabolic fingerprint specific for patients with ACLF (A) and eigenmetabolite of the specific ACLF metabolic cluster (B–D). Data extracted from Ref. [49].

The most interesting aspect of the work by Moreau et al. was the identification of a blood fingerprint composed of 38 metabolites able to discriminate patients with ACLF from those without [49] (Figure 4). The intensity of this metabolomic fingerprint increased across ACLF grades and was similar in patients with kidney failure and in those without, indicating that the fingerprint reflected not only decreased kidney excretion but also altered cell metabolism (Figure 4). Nevertheless, the finding that the higher the plasma levels of inflammatory markers (TNF-α, soluble CD206 and soluble CD163), the higher the intensity of the metabolic alteration positions systemic inflammation as its major driving force [49]. The goal of the intense catabolic metabolism in patients with AD cirrhosis is to provide nutrients to the energetically expensive inflammatory response, which must face the production of inflammatory mediators, immune cell proliferation and migration, respiratory burst, and production of acute-phase proteins [59]. The energetically expensive systemic inflammatory response requires reallocation of stored nutrients to fuel immune activation. To cope with this, immune cells compete with other maintenance programs for energy, including those ensuring proper functioning of peripheral organs. Ultimately, the energetic trade-off between immune activation and organ function homeostasis may lead to peripheral organ hypometabolism and organ dysfunction and failure in these patients.

The ACLF-specific blood metabolite fingerprint also included metabolites that suggest that increased glycolysis in circulating leukocytes is another hallmark of ACLF (Figure 4) [49]. Under normal conditions, mammalian cells obtain the vast majority of energy from mitochondrial oxidative phosphorylation (OXPHOS), which combines electron transport with cell respiration and ATP synthesis [63]. However, in inflammatory conditions, mitochondria become dysfunctional and cells shift from producing ATP by OXPHOS to aerobic glycolysis [63]. Aerobic glycolysis (also known as the Warburg effect) ultimately produces lactate and generates two molecules of ATP per molecule of glucose, and thus is less efficient than OXPHOS, which generates about 36 ATPs per each molecule of glucose [63]. An important aspect to consider is that lactate as an end-product of glycolysis is secreted in high amounts by innate immune cells upon activation, and that this metabolite acts as a negative feedback to limit inflammation by decreasing cytokine production and migration of monocytes and macrophages [64]. Nevertheless, a clear disadvantage of glycolysis is that it highly depends on glucose as a sole fuel source, whereas mitochondrial OXPHOS has more metabolic flexibility and can use fatty acids and amino acids, for example, as carbon sources [63]. Patients with AD cirrhosis also exhibit increased blood levels of intermediates of the pentose phosphate pathway, which branches off from glycolysis at the first committed step of glucose metabolism, suggesting that cytosolic glucose metabolism through alternative routes to glycolysis is also common in these patients [59]. At early stages of injury, energy homeostasis is still attainable through diverting energy production from OXPHOS to glycolysis. However, this is a short-term solution because cells are unable to sustain high energy production from glycolysis at more advanced stages of chronic liver disease [65].

The specific ACLF metabolic fingerprint also contains metabolites indicating depressed mitochondrial ATP-producing fatty acid β-oxidation. In this regard, it is well established that systemic inflammation promotes important changes in mitochondrial function in peripheral organs that preclude appropriate mitochondrial energy production. Indeed, systemic inflammation inhibits both the translocation of fatty acids into the mitochondria and fatty acid β-oxidation and decreases oxidative phosphorylation and ATP production [63]. Consistent with this view, in our study serum levels of hexanoylcarnitine and tetradecenoylcarnitine, which are representatives of medium- and long-chain acylcarnitines and are established markers of incomplete mitochondrial β-oxidation of fatty acids, were remarkably elevated in patients with ACLF (Figure 4) [49].

## The genomic and transcriptomic landscape of ACLF

The intensity of systemic inflammation and the response of the immune system to PAMPs and DAMPs in patients with AD cirrhosis developing ACLF might also be under the influence of host genetic factors. In this regard, certain single-nucleotide polymorphisms (SNPs) have been demonstrated to modulate the release of inflammatory molecules by innate immune cells or the induction of changes in the expression of TLRs. For example, our laboratory screened a panel of SNPs located in six different genes strongly associated with the inflammatory process in a population of 279 patients with AD cirrhosis, of whom 178 presented ACLF. Specifically, we analyzed two SNPs within the IL-1 gene cluster (rs1143623 SNP in the promoter of the gene coding for IL-1β, the key pro-inflammatory cytokine driving the “cytokine storm” and rs4251961, a SNP in the promoter of IL-1ra gene, which inhibits inflammatory response by antagonizing the binding of IL-1 to its receptor); a SNP (rs1800871) in the gene coding for the anti-inflammatory cytokine IL-10 and another SNP (rs4969170) in the suppressor of cytokine signaling (SOCS) 3; the rs31315500 SNP coding for nucleotide-binding oligomerization domain-containing protein 2 (NOD2), a receptor which recognizes bacterial LPS and is primarily expressed in peripheral blood leukocytes; and finally, a SNP (rs1878022) in the chemokine-like receptor 1 (*CMKLR1*) gene, which encodes a G protein-coupled receptor that recognizes RvE1, a small bioactive lipid mediator with potent activity in the resolution of inflammation (see above). Among these SNPs, we identified two polymorphisms belonging to the IL-1 gene cluster (IL-1β and IL-1ra) in strong association with ACLF [66]. The IL-1β SNP was a protective factor against ACLF (OR: 0.34, 95% CI 1.22–5.57, p<0.05) whereas the IL-1ra SNP showed a dycotomic association depending on the inheritance model (overdominant: OR: 0.58, 95% CI 0.35–0.95, p<0.05; codominant: OR: 2.61, 95% CI 1.22–5.57, p<0.05). Notably, a lower frequency of both variants was seen in patients with ACLF (20%) than in those without (80%). The functional role of the IL-1β variant was confirmed by a reduction in plasma IL-1β levels in parallel with the presence of decreased circulating cytokines (i.e., IL-1α, IL-6, granulocyte colony stimulating factor G-CSF and granulocyte/macrophage colony stimulating factor GM-CSF), CRP levels and white blood cell count. The IL-1ra variant was also functional and was associated with changes in IL-ra and circulating cytokine levels in a genotype-dependent manner [66]. Taken together, our study identified two common functional polymorphisms in the IL-1 gene cluster with strong association with the inflammatory process related to the development of ACLF. More recently, it was demonstrated that genetic variants in genes coding for receptors of the innate immune system such as NOD2 or ligands such as mannan-binding lectin (MBL) and MBL-associated serine proteases (MASP) 2 have been reported to associate with increased short-term mortality in patients with AD cirrhosis and ACLF [67].

Although targeted genotype analysis has been instrumental in settling the first evidence of the influence of the genetic background on the clinical course of advanced liver disease, it is essential to obtain a far-reaching genome-wide view to achieve a complete understanding of the genetic architecture of complex phenotypes such as that of ACLF. That is why genome-wide association studies (GWAS) are needed to highlight the value of agnostic approaches in the search for genetic variants with pathophysiological implications. Such a study was recently performed by Tan et al. who carried out GWAS among 399 hepatitis B virus (HBV)-related ACLFs and 401 asymptomatic HBV carriers without antiviral treatment and identified HLA-DR as the major locus for susceptibility to HBV-related ACLF [68]. A more ambitious GWAS project is currently ongoing in our laboratory in more than 3,000 patients with alcohol and/or virus-related AD cirrhosis and ACLF, including patients from different geographic regions and different ancestry. This GWAS project is genotyping more than 750,000 SNPs to provide robust evidence of genetic determinants in the pathogenesis of ACLF.

Transcriptomics has evolved in the last decade as an invaluable tool for investigating changes in immunological conditions, mainly by the identification of modules of highly co-expressed transcripts that identify specific immune cell subsets [69]. Recently, Weiss and collaborators performed whole-blood (“bulk”) transcriptomics in patients with ACLF characterized by ramping blood leukocytosis, neutrophilia, and lymphopenia. These authors identified the upregulation of three modules containing transcripts related to neutrophils and monocytes and the downregulation of five modules containing transcripts related to dendritic cell and lymphocyte activation, memory and effector functions [70]. These findings are consistent with a defective capacity of patients with ACLF to mount effective immune responses and therefore have a higher risk of developing serious and recurrent infections. More recently, our laboratory has exploited transcriptomics to profile the effects of human serum albumin on immune cells from patients with AD cirrhosis and ACLF [71]. The most relevant finding of this investigation was that albumin exerts widespread changes on the immune cell transcriptome, in particular in genes related to the endosomal compartment involved in cytosolic DNA sensing and type I interferon responses [71]. Also, very recently, an elegant integrated transcriptomic and metabolomic analysis together with chromatin immunoprecipitation coupled to DNA sequencing has been performed by Massey and collaborators, allowing these authors to identify that the gene hexokinase domain containing 1 represents a novel biomarker and therapeutic target for patients with alcoholic hepatitis and cirrhosis [72]. By means of RNA sequencing, the same group also identified that defective HNF4a-dependent gene expression is a driver of liver failure in patients with alcohol-related liver disease [73]. Finally, profiling of miRNAs in patients with cirrhosis and ACLF have identified a panel of 11 dysregulated miRNAs associated with organ failure, encephalopathy, bacterial infection and poor outcome [74].

## Conclusions

The ACLF syndrome represents a new paradigm among the diseases characterized by the presence of an excessive systemic inflammatory response associated with organ failure(s). The ACLF syndrome is, thus, a suitable disease condition to investigate the mechanisms underlying systemic inflammation and tissue damage and to advance the identification of the triggers of systemic inflammatory response associated with end-organ dysfunction. The ACLF syndrome is also a suitable condition for the identification of novel biomarkers, and current ongoing studies using cutting edge technologies and integrated multi-omics approaches are rapidly accelerating this process. The identification and clinical validation of these new biomarkers is of utmost importance to guide the discovery of novel therapies/interventions that reduce inflammation without inducing immunosuppression in these patients.
